# Role of the N-Terminal Seven Residues of Surfactant Protein B (SP-B)

**DOI:** 10.1371/journal.pone.0072821

**Published:** 2013-09-02

**Authors:** Mahzad Sharifahmadian, Muzaddid Sarker, Dharamaraju Palleboina, Alan J. Waring, Frans J. Walther, Michael R. Morrow, Valerie Booth

**Affiliations:** 1 Department of Biochemistry, Memorial University of Newfoundland, St. John's, Newfoundland and Labrador, Canada; 2 Department of Medicine at Harbor UCLA, Division of Molecular Medicine, Torrance, California, United States of America; 3 Department of Physics and Physical Oceanography, Memorial University of Newfoundland, St. John's, Newfoundland and Labrador, Canada; 4 Los Angeles Biomedical Research Institute at Harbor-UCLA Medical Centre, Torrance, California, United States of America; 5 Department of Pediatrics, Leiden University Medical Centre, Leiden, The Netherlands; George Washington University, United States of America

## Abstract

Breathing is enabled by lung surfactant, a mixture of proteins and lipids that forms a surface-active layer and reduces surface tension at the air-water interface in lungs. Surfactant protein B (SP-B) is an essential component of lung surfactant. In this study we probe the mechanism underlying the important functional contributions made by the N-terminal 7 residues of SP-B, a region sometimes called the “insertion sequence”. These studies employed a construct of SP-B, SP-B (1–25,63–78), also called Super Mini-B, which is a 41-residue peptide with internal disulfide bonds comprising the N-terminal 7-residue insertion sequence and the N- and C-terminal helices of SP-B. Circular dichroism, solution NMR, and solid state ^2^H NMR were used to study the structure of SP-B (1–25,63–78) and its interactions with phospholipid bilayers. Comparison of results for SP-B (8–25,63–78) and SP-B (1–25,63–78) demonstrates that the presence of the 7-residue insertion sequence induces substantial disorder near the centre of the lipid bilayer, but without a major disruption of the overall mechanical orientation of the bilayers. This observation suggests the insertion sequence is unlikely to penetrate deeply into the bilayer. The 7-residue insertion sequence substantially increases the solution NMR linewidths, most likely due to an increase in global dynamics.

## Introduction

Lung surfactant is a complex of lipids and proteins that lines the air-water interface at the alveolar surface. It is essential for reducing surface tension and preventing alveolar collapse [1], [2]. Lung surfactant has a complex composition and forms dynamic and intricate two dimensional and three dimensional structures, with a monolayer at the interface as well as associated multilayer structures underneath [3]–[7]. Deficiency or inactivation of lung surfactant leads to potentially lethal respiratory disorders such as neonatal respiratory distress syndrome (NRDS) in premature newborns [8]–[10] and acute respiratory distress syndrome (ARDS) in patients with severe injury or illness [11]–[13]. Surfactant replacement therapy has been quite successful in treating NRDS [14], [15]. However, efforts to use replacement surfactant to treat ARDS have not demonstrated improvements in mortality rates thus far [9], [12].

Approximately 90% by weight of surfactant is lipid, mainly phosphocholine (PC) and phosphoglycerol (PG), and 10% is surfactant proteins (SPs) [3], [16]–[18]. SPs are designated by their chronologic order of discovery as SP-A, SP-B, SP-C, and SP-D [19]. SP-A and SP-D are water soluble and important for host defense [20], [21], whereas SP-B and SP-C are smaller, hydrophobic proteins that are critical for reducing surface tension during breathing [22], [23]. SP-B is the only essential protein component of lung surfactant, as evidenced by the lethality of hereditary SP-B deficiency in humans [24], [25] and the lethal effect of knocking out the SP-B gene in mice [26].

SP-B is a highly conserved member of the saposin superfamily of proteins and thus expected to possess 4 to 5 helices [23], [27]. It is found in the lung as a covalently linked homodimer, with 79 amino acid residues in each monomer. Three intramolecular disulfide bonds are formed by six cysteines, and a seventh cysteine forms an intermolecular bridge to stabilize the SP-B homodimer structure. SP-B has a large proportion (52%) of hydrophobic amino acids, and also has cationic characteristics with a net charge of +7 (per monomer) at neutral pH. Its positive charge and highly hydrophobic nature are thought to provide the basis for interactions between SP-B and the negatively charged lipid components of lung surfactant. A number of potential mechanisms for SP-B have been proposed including providing a link between the monolayer and underlying bilayers, stabilizing lipid structures required for lowering surface tension, promoting transfer of lipids into and out of functional lipid structures, and promoting interfacial adsorption of surfactant from the hypophase to the air–water interface [6], [7], [28]–[31]. However, SP-B's three-dimensional structure has not yet been determined, which is an impediment to understanding its detailed mechanism.

In an alternate approach, a number of studies have addressed structure/function relationships in fragments of SP-B that retain a substantial portion of the function of the full length protein [32]–[36]. NMR structures of some of the individual helices of SP-B have been determined [37], [38]. A structure has also been determined for a larger fragment, termed Mini-B, which retains two of SP-B's four helices (residues 8–25 and 63–78) and much of its function, as assessed by measurements in surfactant-deficient rats [39], [40].

The region of SP-B's N-terminus preceding the first helix, termed the “insertion sequence” [41], [42], is of particular interest. The segment of SP-B comprising residues 1–7, Phe-Pro-Ile-Pro-Leu-Pro-Tyr, resembles proline-rich cell-penetrating peptides [43]. Proline is very singular amongst the set of natural amino acids in that it lacks a backbone HN group to form hydrogen bonds, has a ring structure that restricts its allowable backbone dihedral angles, and has a propensity to form special secondary structures, such as polyproline II helix. The functional role of the insertion sequence has been probed in the context of N-terminal peptides of SP-B and it was found that mutation of any of the proline residues led to decreased surface activity [44]. More recently, the insertion sequence has been examined by adding it on to Mini-B, to create a construct of SP-B, termed “Super Mini-B” (SP-B (1–25,63–78)), which retains the N-terminal insertion sequence, two of the four SP-B helices, specifically the N-terminal helix and the C-terminal helix, the two disulfide bonds that help link the helices together, and an overall charge of +7 [41], [42]. The 7 insertion sequence residues, along with the tryptophan at position 9, were proposed to stabilize the formation of “nanosilos”, structures seen by atomic force microscopy imaging of monolayers deposited at high surface pressures [41]. In this study, we further probe the role of SP-B residues 1–7, by comparing the structure and lipid interactions of the construct that includes the insertion sequence, SP-B (1–25, 63–78) (Super Mini-B), with those of a construct lacking this sequence, SP-B (8–25,63–78) (Mini-B), as well as by characterizing the structural features of SP-B (1–7) by itself.

## Materials and Methods

### Materials

SP-B (1–25,63–78) (FPIPLPYCWLCRALIKRIQAMIPKGGRMLPQLVCRLVLRCS), SP-B (8–25,63–78) (CWLCRALIKRIQAMIPKGGRMLPQLVCRLVLRCS) and SP-B (1–7) (FPIPLPY) were prepared by solid phase synthesis using Fmoc (O-fluorenylmethyl-oxycarbonyl) chemistry as in [41]. The peptides were purified using reversed-phase HPLC and the masses were confirmed by MALDI-TOF spectroscopy. Purity of the sample was determined to be ≥95% by analytical HPLC. Sodium-dodecylsulfate-d_25_ was purchased from Cambridge Isotope Laboratories (Andover, MA) and sodium-dodecylsulfate and sodium azide from Sigma-Aldrich. The phospholipids 1-palmitoyl (*d*_31_)-2-oleoyl-*sn*-glycero-3-phosphocholine (POPC-d_31_) and 1-palmitoyl-2-oleoyl-*sn*- glycero-3-[phospho-rac-(1-glycerol)] (sodium salt) (POPG), were purchased from Avanti Polar Lipids, Inc.

### Solid-state NMR

^2^H NMR was used to observe the perturbation of mechanically-oriented phospholipid bilayers by SP-B (1–25,63–78). To prepare oriented samples, 1 mol% peptide, if present, was co-dissolved with 4 mg of a 7∶3 (molar ratio) mixture of POPC-*d*_31_ and POPG in a mixture of CH_3_OH/CHCl_3_ (1∶1 by volume). The solution, comprising a total volume of 250 μl, was spread onto 12 mica plates (12 mm by 12 mm) by depositing ∼1 μl at the centre of each plate, allowing 3–5 minutes for spreading and drying, and then repeating until the deposition of the full volume was complete. The films on the mica plates were then dried for 2 hours in a fume hood after which residual solvent was removed by exposure to vacuum for ∼8 hours. Films were then hydrated by spreading 5 μL of deuterium-depleted water onto each plate and then leaving the plates in a hydration chamber, along with saturated ammonia phosphate solution, at 4°C for 2 days. The plates were then carefully stacked, wrapped with plastic film, and sealed in heavy plastic wrap. Samples were stored at 4°C before the NMR experiments.

^2^H-NMR spectra were obtained using a locally-assembled spectrometer operating at 61.4 MHz. The oriented samples were positioned in a flat coil (15 mm ×15 mm ×3 mm) with the bilayer normal parallel to the magnetic field of a 9.4 T superconducting solenoid. Spectra were derived from free-induction decays obtained by averaging 60000 transients accumulated using a quadrupole echo sequence [45] with a π/2 pulse length of 4.1 μs and 30 μs pulse separation. Transients were acquired using a digitizer dwell time of 1 μs and oversampling [46] by a factor of 4 to give an effective dwell time of 4 μs. All ^2^H-NMR spectra were acquired at 23°C.

The ^2^H-NMR spectrum of POPC-*d*_31_ in a liquid crystalline bilayer oriented with its normal at an angle β with respect to the applied magnetic field is a superposition of spectral doublets with quadrupole splittings given by



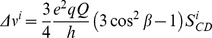
where 
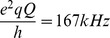
 is the quadrupole coupling constant for carbon-deuterium bonds and *S^i^_CD_* is the orientational order parameter for deuterons on the acyl chain methylene group denoted by *i*. For a given carbon-deuterium bond, the orientational order parameter is given by



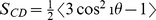
where θ is the angle between the direction of the carbon-deuterium bond and the bilayer normal which is the symmetry axis for fast, axially symmetric reorientation of the lipid acyl chain in the liquid crystalline phase. The average is over all motions and chain conformational changes that modulate the quadrupole interaction on the approximately 10^−5^ s timescale of the ^2^H-NMR. For a saturated phospholipid acyl chain, the orientational order parameter is largest at the headgroup end of the chain, where motions are most constrained, and decreases with position along the chain to the methyl group near the bilayer centre where motions are least constrained. The dependence of orientational order parameter on position along the chain is characterized by the orientational order parameter profile [47], [48].

For a mechanically-oriented sample with bilayer normal parallel to the magnetic field, β = 0° and the spectrum is a superposition of sharp doublets with quadrupole splittings given by
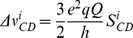



For unoriented bilayers, such as in a multilamellar vesicle sample, the bilayer normal directions are spherically distributed and the spectrum is a superposition of Pake doublets with prominent edges, for each position along the chain, corresponding to β = 90° and thus half the splitting of the corresponding doublet in the oriented bilayer spectrum.

### 2.2 Circular dichroism (CD)

The same peptide samples were used for solution NMR and for CD. SP-B (1–25,63–78), SP-B (8–25,63–78) and SP-B (1–7) in SDS micelles were prepared by dissolving 1 mM peptide and 150 mM deuterated SDS (98% deuterated, Cambridge Isotope Laboratories) in 90% H_2_O and 10% D_2_O. The sample also included 0.2 mM 2,2-dimethyl-2-silapentane-5-sulfonate (sodium salt, DSS) and 0.2 mM sodium azide. The pH was adjusted to 5.0. The CD spectra were acquired on a Jasco J–810 spectropolarimeter (Applied Photophysics, UK). The absorbance at 222 nm was checked for each sample to ensure that it did not exceed an optical density of 1.0. A 1 mm cell was used and spectra were collected between wavelengths of 200 and 260 nm at 308 K. Baselines were established using the appropriate buffer and the means of 4 spectra were analyzed. Secondary structure content was calculated from the spectra using CDPro software (http://lamar.colstate.edu/~ssreeram/CDPro) developed by Woody and co-workers (Sreerama and Woody, 1993). CD values were converted to mean residue ellipticity (MRE). Basis set 2 of the CDPro software was used and analysis was performed using CONTIN/LL method [49].

### Solution NMR

The same samples used for the CD observations of SP-B (1–25,63–78) were also employed in the solution NMR experiments. Data was acquired on a Bruker Avance 600 MHz spectrometer equipped with z-gradients and a triple-resonance TXI probe. The 1D ^1^H experiments were run with 32 transients, and a recycle delay of 1 second, with watergate water suppression, at 37 and 45°C. 2D-TOCSY experimental parameters included a mixing time of 80 ms, acquisitions of 96 transients for SP-B (1–25,63–78) and 128 transients for SP-B (8–25,63–78), with 1024 points in the direct dimension, 480 points in the indirect dimension, and water-gate water suppression. 2D-NOESY experiments were run with a mixing time of 200 ms, 160 transients for SP-B (1–25,63–78) and 128 transients for SP-B (8–25,63–78), 1024 points in the direct dimension, 512 points in the indirect dimension, and water-gate water suppression. The chemical shifts were referenced with respect to an internal DSS standard (0.0 ppm). The data was processed with iNMR (http://www.inmr.net) and analyzed using Sparky [50].

Diffusion-ordered spectroscopy (DOSY) experiments were performed on the same Bruker Avance 600 MHz spectrometer employing pulsed field gradient (PFG) NMR [51] with the same SP-B (1–25, 63–78) SDS sample. The pulse sequence used a stimulated echo with bipolar gradient pulses and one spoil gradient [52] followed by a 3–9–19 pulse for water suppression [53]. The ^1^H signals were attenuated to ∼5% of their initial amplitudes by increasing the gradient strength from ∼2% to 95% in 32 steps. Experiments were performed at 37°C. The pseudo 2D DOSY spectra were processed using iNMR and the diffusion constants extracted using the DECRA package of DOSYToolbox [54].

## Results

### ^2^H NMR of SP-B (1–25,63–78) in oriented lipid bilayers

**Figure 1:**
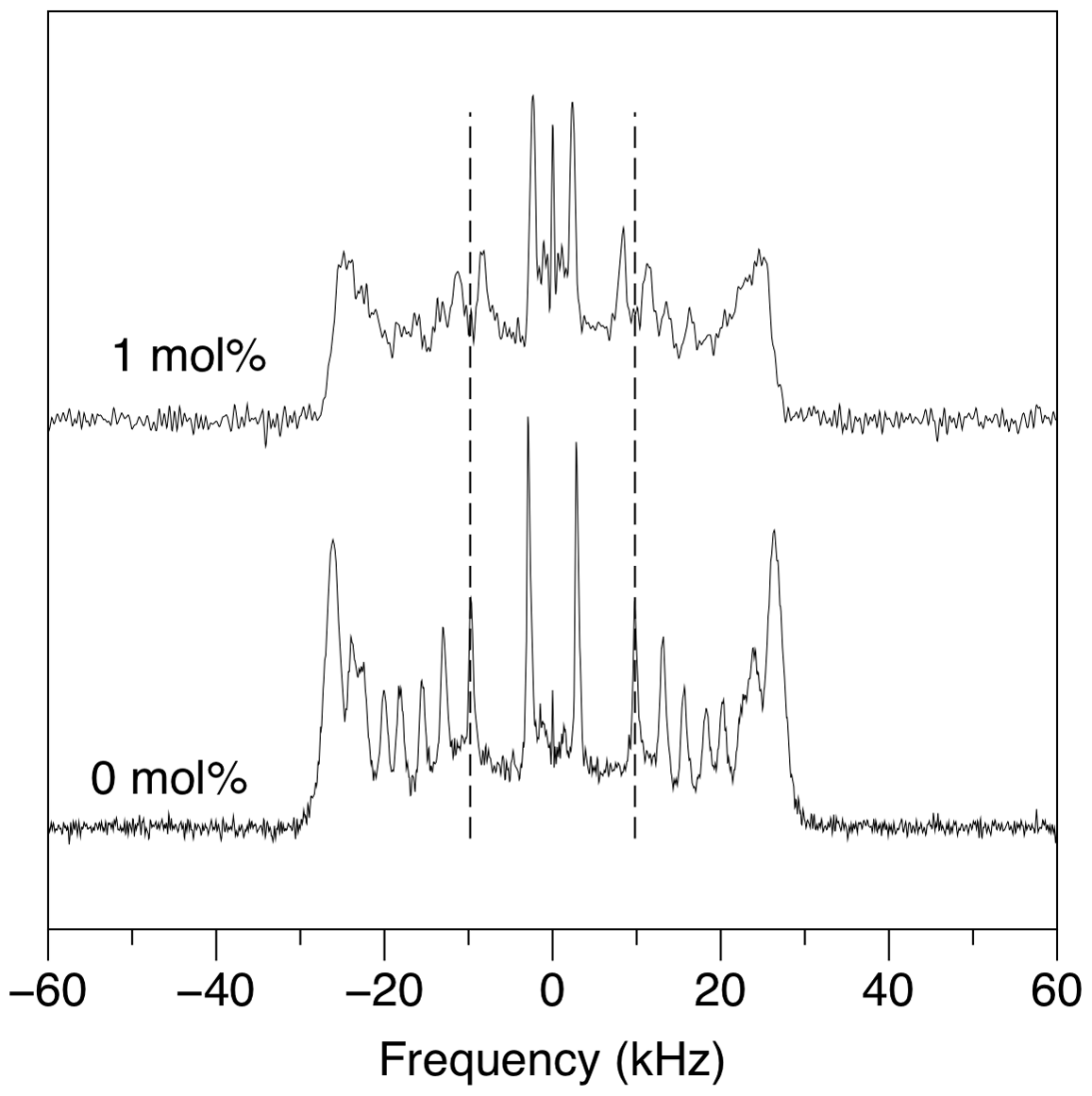
^2^H spectra of mechanically oriented 7∶3 POPC-*d*_31_:POPG bilayers in the presence (upper panel) and absence (lower panel) of 1 mol% SP-B (1–25,63–78). The spectra were acquired with 60000 transients at 23°C. Dashed vertical lines indicate the quadrupole splitting of deuterons on the C15 acyl chain segment of POPC-*d*_31_ in the absence of SP-B (1–25,63–78).

The effects of SP-B (1–25,63–78) on lipid bilayer orientation and chain orientational order were investigated by ^2^H NMR. **Figure 1** shows spectra of POPC-*d*_31_/POPG (7∶3) with and without SP-B (1–25,63–78). In the absence of peptide, the doublets are sharp and well resolved, indicating that the lipid bilayers are well aligned. With the incorporation of 1 mol% SP-B (1–25,63–78), the distribution of spectral area across the spectrum shifts in a way that suggests the emergence of a weak spectral component corresponding to a more random distribution of bilayer normal directions - i.e. Pake quadrupole doublets split by half of the corresponding oriented sample doublet splittings. Doublets in the oriented component of the spectrum also broaden in a way consistent with increased mosaic spread [55]. The presence of SP-B (1–25,63–78) thus appears to disrupt the mechanical orientation in a small fraction of the bilayer material.

**Figure 2:**
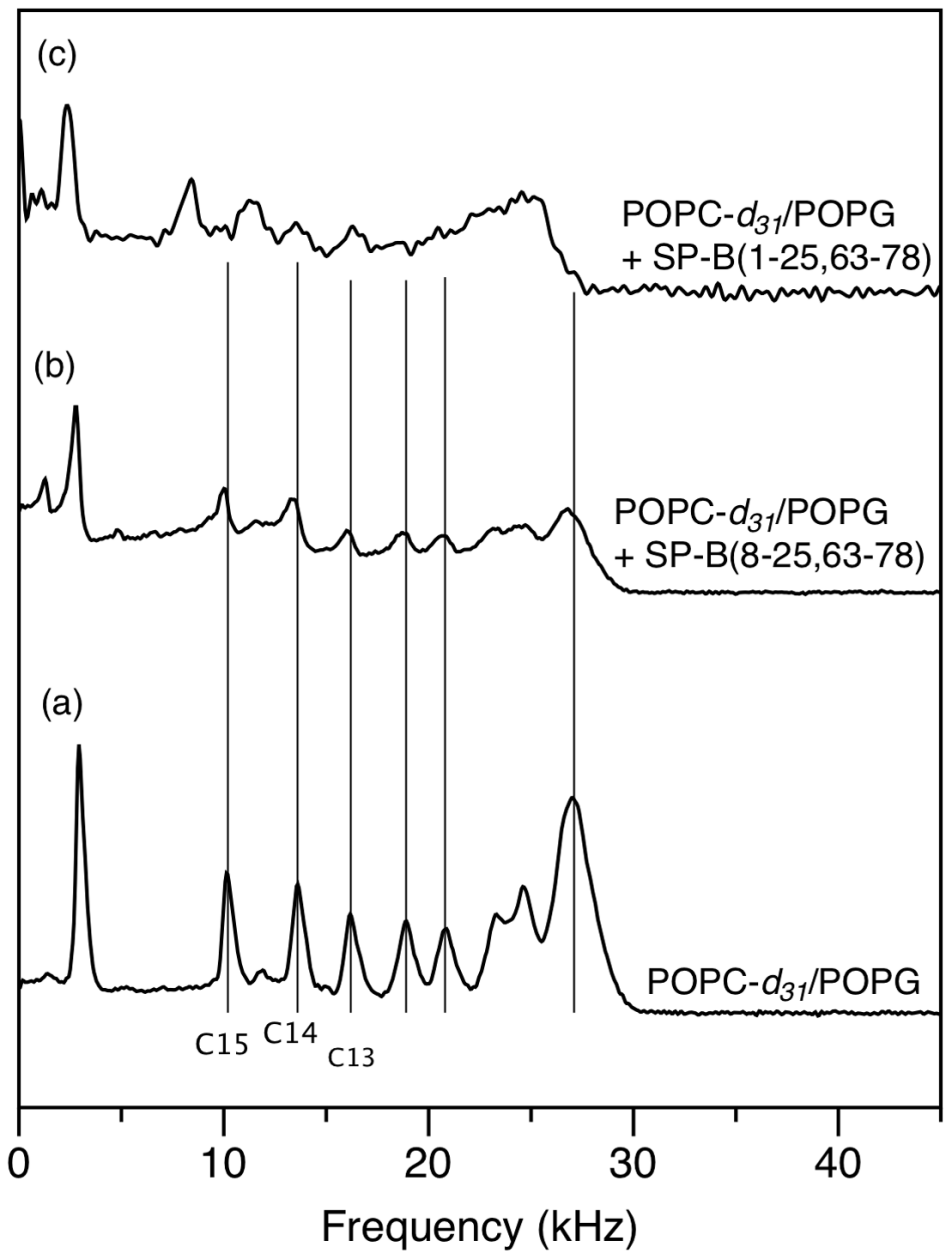
Half ^2^H NMR spectra of mechanically oriented 7∶3 POPC-*d*_31_:POPG bilayers in the absence of peptide (a), in comparison to (b) with 1 mol% SP-B (8–25,63–78) or (c) SP-B (1–25,63–78). The spectra were all acquired at 23°C. Vertical lines indicate the quadrupole splitting of POPC-*d*_31_ deuterons in the absence of peptide.

More conspicuous, however, is the effect of SP-B (1–25,63–78) on the quadrupole splittings, and hence chain orientational order, of that portion of the lipid bilayer material that does remain oriented. The vertical lines in **Figure 2** indicate the quadrupole splitting of deuterons of POPC-*d*_31_ in the absence of SP-B (1–25,63–78). The quadrupole splitting for the C15 deuterons, just adjacent to the methyl group at the tail end of the lipid chains, as well as the C14 and C13 deuteron splittings, are substantially reduced in the presence of SP-B (1–25,63–78) (**Figure 2c**). While more difficult to resolve, the largest quadrupole splittings, corresponding to deuterons in the so-called plateau region near the headgroup end of the acyl chain, are also reduced by the presence of the SP-B (1–25, 63–78). The splittings and corresponding order parameters are quantified in **Table 1**. SP-B (1–25,63–78) is thus seen to increase orientational disorder along the entire lipid acyl chain and particularly near the centre of the bilayer.

**Table 1:** Splittings, Δ ν, and corresponding order parameters, S_CD_, for the resolved peaks of the 2H NMR spectra of POPC-*d*_31_/POPG (7∶3) (Figure 1) in the absence and presence of SP-B (1–25,63–78) (Super Mini-B).

	C15		C14		C13		Plateau	
	Δ ν (kHz)	S_CD_	Δ ν (kHz)	S_CD_	Δ ν (kHz)	S_CD_	Δ ν (kHz)	S_CD_
Without SP-B (1–25,63–78)	19.8±0.8	0.079	26.4±0.9	0.11	31.4±0.9	0.13	52.5±1.2	0.21
With SP-B (1–25,63–78)	16.7±0.8	0.066	22.5±0.9	0.090	26.7±0.9	0.11	50.3±1.2	0.20

The splittings quoted derive from a single experiment; however the uncertainty has been estimated based on the standard deviation in splittings derived from 5 separate control experiments with the same lipid composition.

### Structural Characteristics of SP-B (1–25,63–78) in SDS micelles

**Figure 3:**
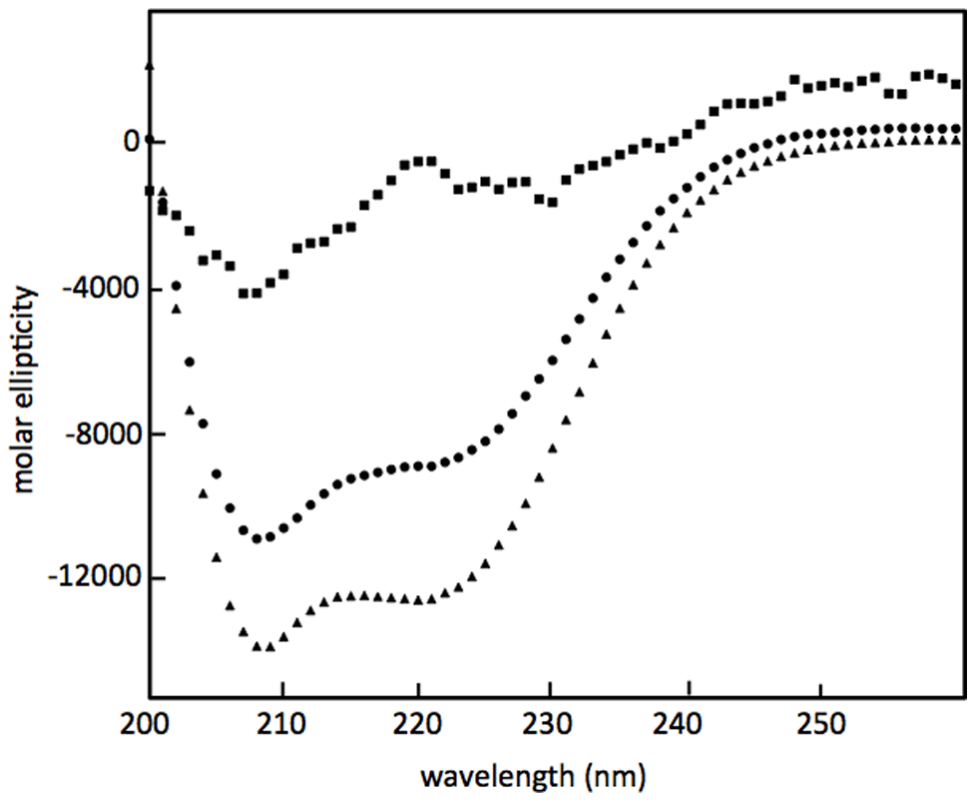
A) Far-UV CD spectra of SP-B (8–25,63–78) (triangles), SP-B (1–25,63–78) (circles) and SP-B (1–7) (squares) dissolved in SDS micelles. Spectra were taken using a 1 mm path-length quartz cuvette from 200 nm to 260 nm at 25°C. Shown are single scans. Three additional spectra of each sample were acquired and the 4 scans averaged together before % secondary structure was extracted (**Table 2**).

Circular dichroism (CD) experiments were carried out with SP-B (1–25,63–78) in SDS micelles, and, for comparison, with SP-B (8–25,63–78) and SP-B (1–7) in SDS micelles (**Figure 3**). The secondary structure percentages were calculated from the spectra using two different methods, ContinLL with reference protein set SP22X [49] which fits the CD data using 5 types of secondary structure, as well as K2D [56], which uses only three secondary structure types in the fit (**Table 2**). Both methods indicate that SP-B (1–25,63–78) possesses a smaller fraction of helical structure and a greater fraction of unstructured residues, compared to SP-B (8–25,63–78). The decrease in the fraction of helical structure in the longer peptide is as expected given that the solution NMR data indicate no major structural changes in the helical portion of the fragments with the inclusion of the extra 7 residues in SP-B (1–25,63–78) (see below). Since NMR gives residue-specific information on secondary structure and CD reports on the fraction of residues in a particular structure, both results are consistent with the same number of residues taking on a helical structure in the two peptides. However, the CD data provide no clear indication of the structural preferences of these extra 7 residues, which is consistent with the known difficulties in extracting anything but the fraction of helical structure from CD spectra of this wavelength range [57]. The ContinLL deconvolution suggests the additional residues in SP-B (1–25,63–78) take on mainly a mix of unordered and β-sheet conformations, but the K2D analysis is more consistent with the extra residues forming exclusively unordered structures. A peptide consisting of SP-B (1–7) by itself was also subjected to CD analysis in both SDS micelles (**Figure 3**) and in 40% hexafluoroisopropanol (HFIP) (data not shown). However, the peptide was poorly soluble and the secondary structure deconvolution unreliable due to the poor signal-to-noise in the spectrum and the difficulties in reliably determining the effective concentration, and thus the deconvolution data has not been included in the analysis.

**Table 2:** Percent secondary structure content of SP-B (1–25,63–78) (Super Mini-B), SP-B (8–25,63–78) (Mini-B) and SP-B (1–7) (the insertion sequence) in SDS micelles calculated from CD spectra (average of 4 scans).

Secondary Structure (%)	*α*-helix	3_10_-helix	*β*-Sheet	Turn	Polyproline II	Unordered
SP-B (1–25,63–78) (Super Mini-B) *ContinLL Method*	24	7	13	14	6	36
SP-B (1–25,63–78) (Super Mini-B) *K2D Method*	29	–	16	–	–	55
SP-B (8–25,63–78) (Mini-B) *ContinLL Method*	37	9		13	5	30
SP-B (8–25,63–78) (Mini-B) *K2D Method*	42	–	16	–	–	41

Deconvolution of CD spectra was accomplished using CDPro, with basis set 2 and the CONTIN/LL algorithm [49] and also with the K2D method [52].

**Figure 4:**
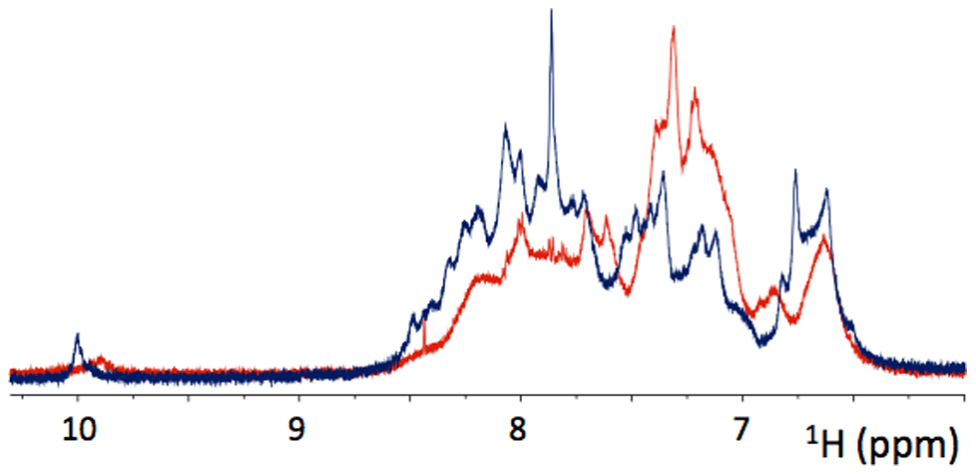
HN region of the ^1^H NMR spectra of 1 mM SP-B (1–25,63–78) in SDS micelles at 45°C (red) and 1.5 mM SP-B (8–25,63–78) in SDS micelles at 37°C (blue). The number of transients was 32. The intensity scale is arbitrary but has been normalized to take into account the differences in peptide concentration.

Solution NMR experiments were also used to assess the conformation of SP-B (1–25,63–78) in SDS micelles. 1D proton, 2D-TOCSY and 2D-NOESY NMR experiments were acquired at several temperatures. 45°C was chosen for analysis because this temperature provided the greatest peak intensity and resolution. The HN region of the 1D ^1^H NMR spectrum of SP-B (1–25,63–78) in SDS micelles at 45°C (**Figure S1**) is shown along with a spectrum of SP-B (8–25,63–78) in SDS micelles at 37°C (**Figure 4**). The overall dispersion of peaks in the HN region is similar, implying a similar level of structuring for both peptides. However in the SP-B (1–25,63–78) spectrum the peaks appear broader.

**Figure 5:**
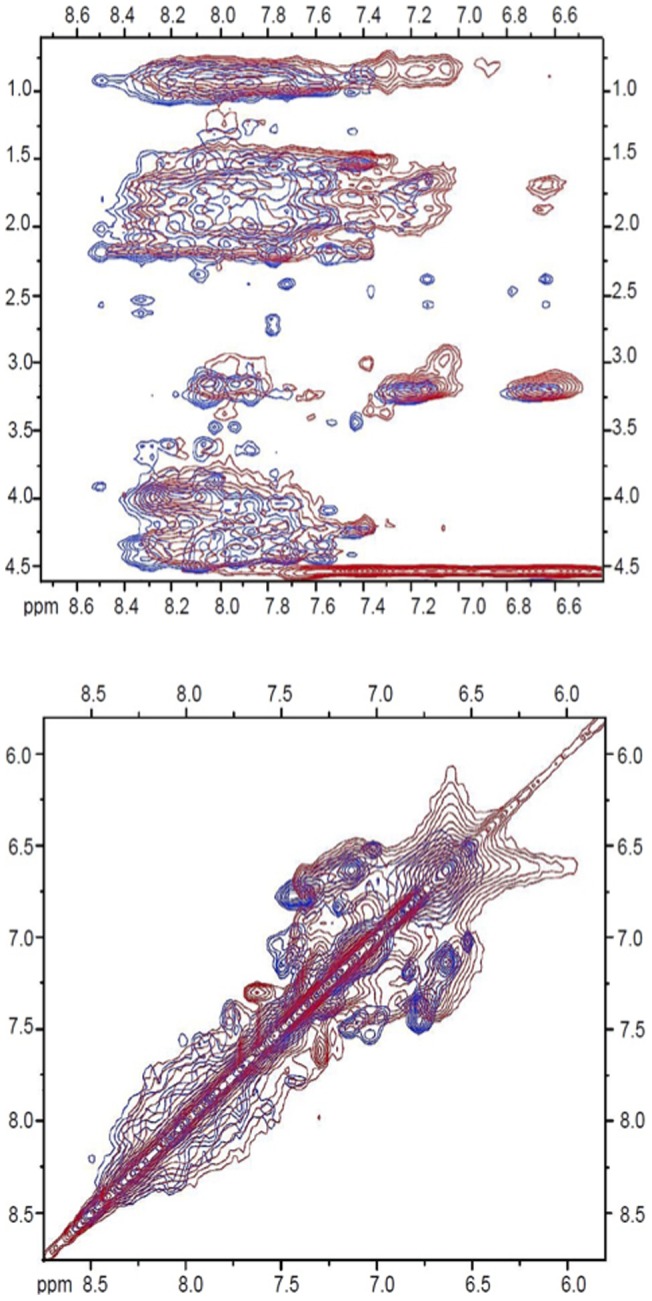
Selected regions of 2D NOESY NMR spectra of 1 mM SP-B (1–25,63–78) in 150 mM SDS solution at pH 5 and 45°C (red) and 1 mM SP-B (8–25,63–78) in 150 mM SDS at pH 5 and 37°C (blue).

2D TOCSY (**Figure S2**) and NOESY (**Figure S3**) NMR spectra were acquired for the SP-B (1–25,63–78)/SDS samples. In **Figure 5**, selected regions of the NOESY spectra are displayed overlaid on previously acquired spectra of SP-B (8–25,63–78) [40]. As in the 1D NMR spectra, the peaks in the 2D spectra of SP-B (1–25,63–78) are broad compared to those from SP-B (8–25,63–78). Given the unique amino acid composition of the insertion sequence, it was possible to use the SP-B (1–25,63–78) spectra, along with spectra of SP-B (1–7) acquired under identical conditions (**Figures S4 and S5**), to obtain partial resonance assignments for this region (**Table 3**). The peaks for the regions outside residues 1–7 in the SP-B (1–25,63–78) spectra overlay reasonably well with the strong peaks in the SP-B (8–25,63–78) spectra. In particular, the strong HN-HN peaks that are indicative of α-helical structure [58] are retained in the SP-B (1–25,63–78) spectra. Overall the similarity in peak positions between the two peptides indicates that the presence of the insertion sequence does not modify the structure of the remainder of the peptide, at least not substantially enough to detect given the differences in conditions and lineshape between the two spectra.

**Table 3:** Partial ^1^H chemical shift assignments (in ppm) for residues 1–7 of SP-B, as assigned based on 2D TOCSY and NOESY NMR experiments with SP-B (1–7) and SP-B (1–25,63–78) (Super Mini-B) in deuterated SDS at 45°C.

Residue	NH	Hα	Hβ	H_other_
F1	7.66	4.52	3.45, 3.24	Hδ = 7.67, Hε = 7.32 Hζ = 7.55
P2/4/6	N/A	3.85	1.78, 2.12	Hγ = 1.29,1.55, Hδ = 3.66,3.18,
P2/4/6	N/A	4.38	1.77, 2.28	Hγ = 1.94,1.78, Hδ = 3.47,3.24
P2/4/6	N/A	4.53	2.04, 2.14	Hγ = 1.54,1.16, Hδ = 3.79,3.45
I3	unassigned	3.65	1.74	Hγ2 = 0.99
L5	7.31	4.52	2.05, 2.22	Hγ = 1.91, Hδ = 0.94,0.95
Y6	7.10	4.51	3.07, 2.74	Hδ = 7.10, Hε = 6.96

Note that although three sets of proline resonances were identified, none of them could be identified with a particular proline in the sequence.

There are two possible explanations for the greater linewidth of 1D and 2D NMR spectra of SP-B (1–25,63–78) compared to the spectra from SP-B (8–25,63–78). One is that there is an increase in the size of the SP-B (1–25,63–78)/SDS micelle complexes being observed, compared to the complexes formed with SP-B (8–25,63–78). Alternatively, the broader lines could derive from conformational fluctuations on the millisecond to microsecond timescale [59].

### Peptide Self Association

**Figure 6:**
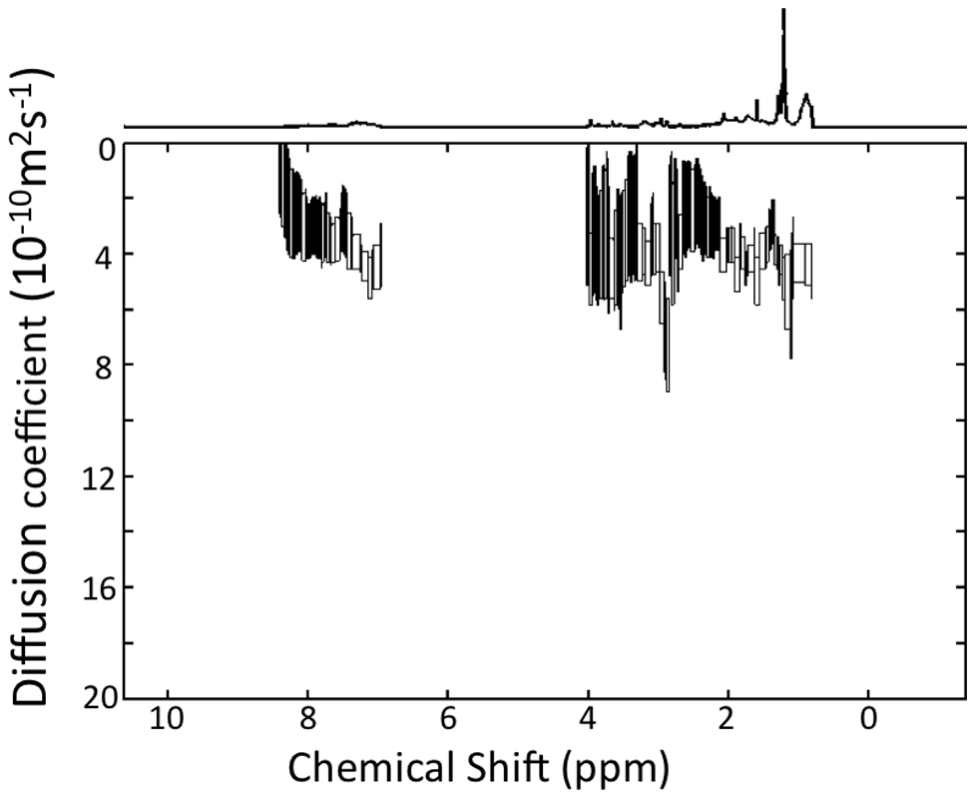
DOSY NMR analysis of SuperMini-B in SDS micelles at 37°C.

In order to explore the possibility that there is a substantial increase in the size of SP-B (1–25,63–78) complexes, we used diffusion NMR spectroscopy to measure the apparent translational diffusion constant at 37°C. As seen in **Figure 6**, the diffusion constants observed in the HN/aromatic region, and thus deriving from the peptide, are in the range of 3 to 4×10^−10^ m^2^s^−1^. The apparent diffusion constants derived from the detergent peaks are similar. The apparent diffusion constant measured previously for SP-B (8–25,63–78) in SDS micelles at the same temperature was 2.7±0.3×10^−10^ m^2^s^−1^ [60]. Thus, the diffusion measurements imply that SP-B (1–25,63–78) is in similar sized complexes or slightly smaller complexes compared to SP-B (8–25,63–78). To further test the possibility that the increase in linewidth might derive from an increase in self-association of the peptide, we performed titration experiments in which 1D NMR spectra of SP-B (1–25,63–78) were acquired at increasing peptide concentrations (data not shown). No changes in the spectra with peptide concentration were observable.

## Discussion

To explore the role of the N-terminal 7 residues of SP-B, we first used ^2^H NMR to examine the effect of SP-B (1–25,63–78) on mechanically oriented lipid bilayers composed of a mixture of zwitterionic and anionic phospholipids, POPC-*d*_31_/POPG (7∶3) (**Figure 1**). The presence of SP-B (1–25,63–78), a fragment which contains the N-terminal 7 residues as well as the first and last helices of SP-B, caused a substantial effect on the ^2^H NMR splittings and hence acyl chain orientational order, particularly deep in the bilayer (**Table 1**). The magnitude of the SP-B (1–25,63–78)-induced effect was particularly striking when compared to the effect of SP-B (8–25,63–78) [55], i.e. an identical peptide except for the absence of residues 1–7. 1 mol% SP-B (8–25,63–78) induced only a slight decrease in splitting (**Figure 2**), compared to the much more marked effect of 1 mol% SP-B (1–25,63–78). More quantitatively, the order parameters, S_CD_, calculated from the splittings (Table 1) for the acyl chain positions just adjacent to the terminal methyl group decreased with the addition of 1 mol% SP-B (1–25,63,78) by 18%, 20% and 17% for C15, C14, and C13, respectively. On the other hand, the decrease in the same order parameters with 1 mol% SP-B (8–25,63,78) were less than 1%. In earlier studies, we also measured the reduction in POPC/POPG-*d*_31_ order parameter induced by the C-terminal helix alone [61]. The lowest peptide concentration studied in that work was 2.4 mol% SP-B (63–78) and this reduced the order parameters calculated for positions C13, C14, and C15 of POPC-d_31_ by 7.4%, 7.5%, and 6.3% respectively - i.e. less than half the disruption of SP-B (1–25,63–78) with more than twice the peptide. Thus, it appears that residues 1–7 of SP-B cause a dramatic decrease in acyl chain orientational order, especially towards the centre of the bilayer.

The ability of the N-terminal 7 residues of SP-B to disrupt order deep within the bilayer could be an important component of SP-B's suggested function in mediating transfer of lipids between the surfactant monolayer at the surface and the underlying multilayers. Mutation of the N-terminal prolines to alanines in SP-B (1–25) leads to less effective reinsertion of surface-active material into the expanding interface film [44] and deletion of the insertion sequence from SP-B (1–25), reduces this peptide's ability to promote the formation of a fluid isotropic phase in the lipids [62].

When a peptide substantially reduces the orientational order of the lipid chains, in particular deep in the bilayer, this indicates that the peptide locates close to the polar/apolar interface [61], [63]. This is because a peptide positioned close to the lipid head groups can increase the spacing between lipids without constraining the motions of the acyl chains, that, with the increase in headgroup spacing, have more orientational freedom. By contrast, peptides that insert deeply into the bilayer have little effect on acyl chain order [63], [64] because the peptide itself confers stearic constraints on acyl chain motions. That Super Mini-B (SP-B (1–25,63–78)) is so effective at disrupting the order deep in the bilayer interior is thus an indication that the N-terminal 7 residues likely do not penetrate deeply enough to constrain the amplitude of lipid acyl chain reorientation. This is not because the 7 residues cannot form a long enough structure; for an extended conformation such as a β-strand, the translation per residue is about 3.5 Å [65] and so 7 residues could, in theory extend out to 24.5 Å, which is close to the width of the hydrophobic region of a lipid bilayer. Instead, it appears that the insertion sequence residues preferentially position closer to the surface of the bilayer. Consistent with this interpretation, fluorescence quenching experiments found that residues 1–6 of SP-B (1–25) locate near the bilayer surface, while residues 7–9 insert more deeply [66]. 2D TOCSY and NOESY solution NMR experiments indicate that the 7 N-terminal residues do not induce a major change in the structure of the helical regions of the protein. This is inferred by the absence in major changes in the positions of the peaks from SP-B (8–25,63–78) in the context of SP-B (1–25,63–78) (**Figure 5**). This NMR result backs up previous studies in which FTIR spectra indicated little difference in helical content between SP- B (8–25,63–78) and SP-B (1–25,63–78) [42].

Despite the apparent absence of alterations in helicity, the solution NMR spectra of SP-B (1–25,63–78) (**Figures 4** **and** **5**) did exhibit an increase in linewidth compared to spectra of SP-B (8–25,63–78) consistent with an increase in the size of the peptide/SDS micelle and/or an increase in dynamics on an intermediate timescale. Dimerization of SP-B (1–25,63–78) was previously suggested on the basis of SDS-PAGE electrophoresis [42]. However, diffusion NMR measurements (**Figure 6**) indicate that the size of the SP-B (1–25,63–78)/SDS complexes is similar to that previously measured for SP-B (8–25,63–78) complexes and titration experiments also failed to provide evidence of peptide self-association. Thus, the most likely explanation for the increase in linewidth is a change in the global dynamics of SP-B (1–25,63–78) compared to SP-B (8–25,63–78). An increase in dynamics on the millisecond to microsecond timescale would account for the increase in line widths [59] and would also be consistent with the increase in motional flexibility of the lipid chains observed in the ^2^H-NMR experiments.

The insertion sequence contains three proline residues and thus we wanted to explore the possibility that a polyproline helical structure is formed. The circular dichroism data does not clearly indicate what type of structure this 7-residue piece forms – although as expected the CD spectra do exclude α- or 3_10_ helix **(** **Table 2** **,** **Figure 3**). The CD spectra are not inconsistent with the 7 residues taking on a polyproline helical type secondary structure in that while the spectrum of SP-B (8–25,63–78) exhibits a canonical helical shape, with minima at 209 nm and 220 nm, the spectrum of SP-B (1–25,63–78) displays an altered shape with a slight shift of the 209 minimum to a shorter wavelength and a less pronounced minimum at 220 nm. However, the deconvolution of the CD spectra (**Table 2**) suggests the insertion sequence could also consist of a large portion of β and/or unordered structures.

In summary, we report that the 7-residue insertion sequence of SP-B does not substantially disrupt the helical region of the peptide, but does cause alterations in the solution NMR line-width, most likely via alterations in the global mobility of the peptide We also find that the inclusion of the insertion sequence leads to dramatic increases in acyl chain disorder in the centre of the bilayer, as compared to the more modest degree of disorder produced by SP-B peptides lacking the insertion sequence. This degree of acyl chain disruption is consistent with a location of the insertion sequence close to the bilayer surface and is likely important in promoting the bilayer disruptions important to SP-B's role in adsorption and re-spreading of lung surfactant.

## Supporting Information

Figure S1 **Full ^1^H NMR spectrum of 1 mM SP-B (1–25,63–78) in SDS micelles at 45°C.**
(TIFF)

Figure S2 **2D TOCSY NMR spectrum of 1 mM SP-B (1–25,63–78) in 150 mM SDS solution at pH 5 and 45°C.**
(TIFF)

Figure S3 **Full 2D NOESY NMR spectrum of 1 mM SP-B (1–25,63–78) in 150 mM SDS solution at pH 5 and 45°C.**
(TIFF)

Figure S4 **2D NOESY NMR spectrum of SP-B (1–7) in 300 mM SDS solution at pH 5 and 45°C.**
(TIFF)

Figure S5 **2D TOCSY NMR spectrum of SP-B (1–7) in 300 mM SDS solution at pH 5 and 45°C.**
(TIFF)
