# Supplementary figures and images for: Role of the N-Terminal Seven Residues of Surfactant Protein B (SP-B)
Source: PLoS One. 2013 Sep 2;8(9):e72821. doi: 10.1371/journal.pone.0072821 (PMC3759391; doi:10.1371/journal.pone.0072821)

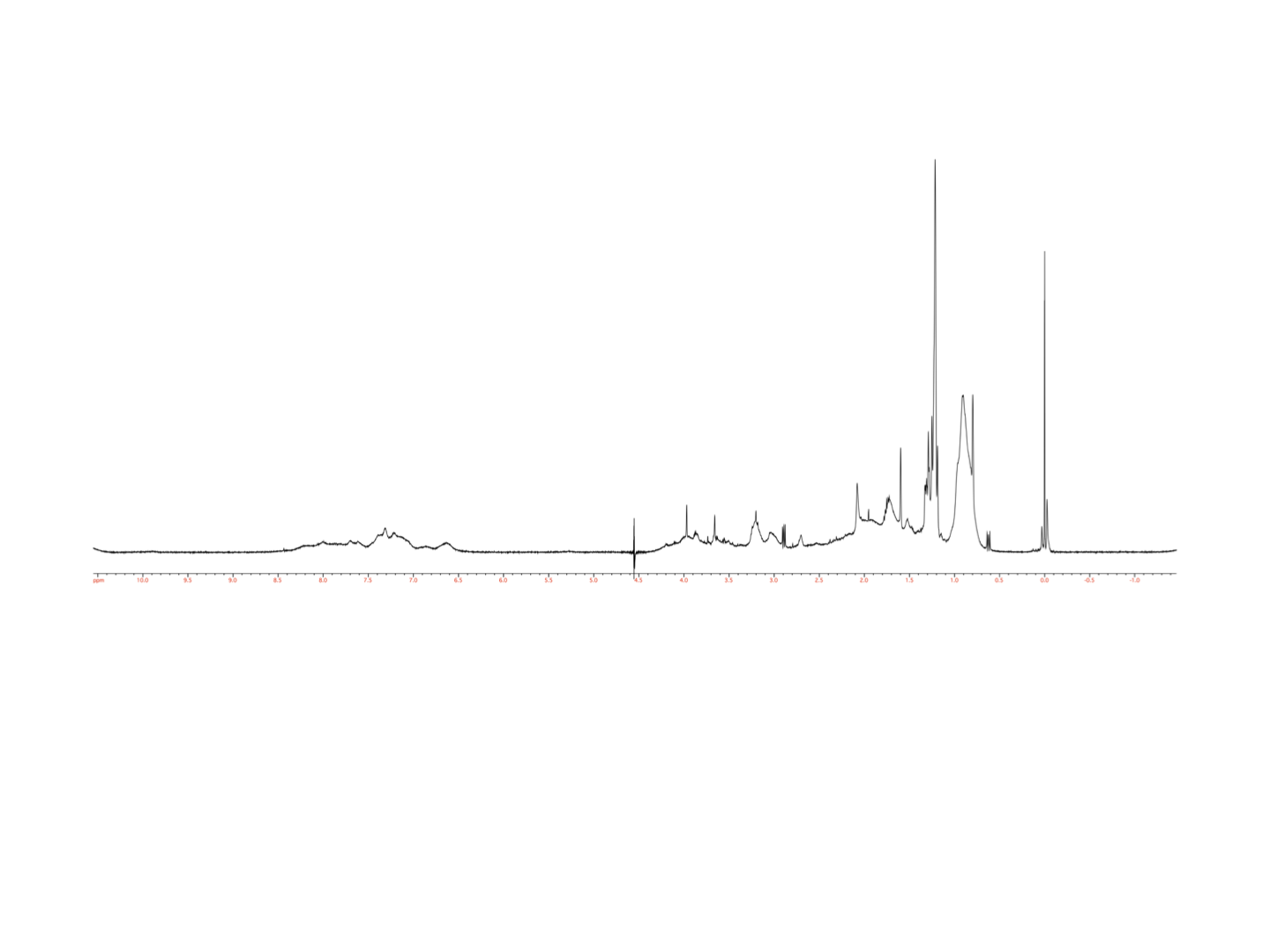

Supplement: Figure S1 — Full 1H NMR spectrum of 1 mM SP-B (1–25,63–78) in SDS micelles at 45°C. (TIFF) [file pone.0072821.s001.tiff]

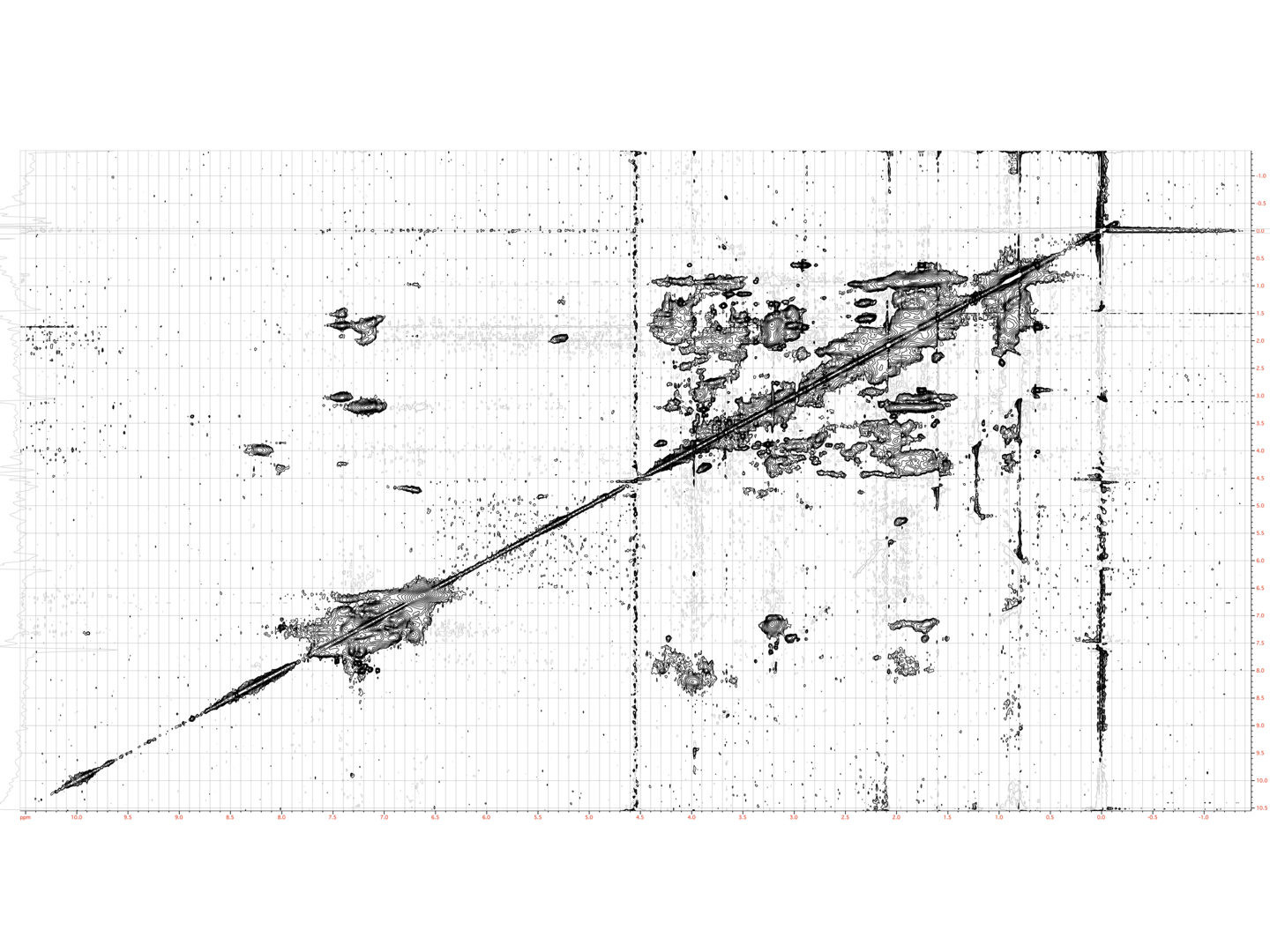

Supplement: Figure S2 — 2D TOCSY NMR spectrum of 1 mM SP-B (1–25,63–78) in 150 mM SDS solution at pH 5 and 45°C. (TIFF) [file pone.0072821.s002.tiff]

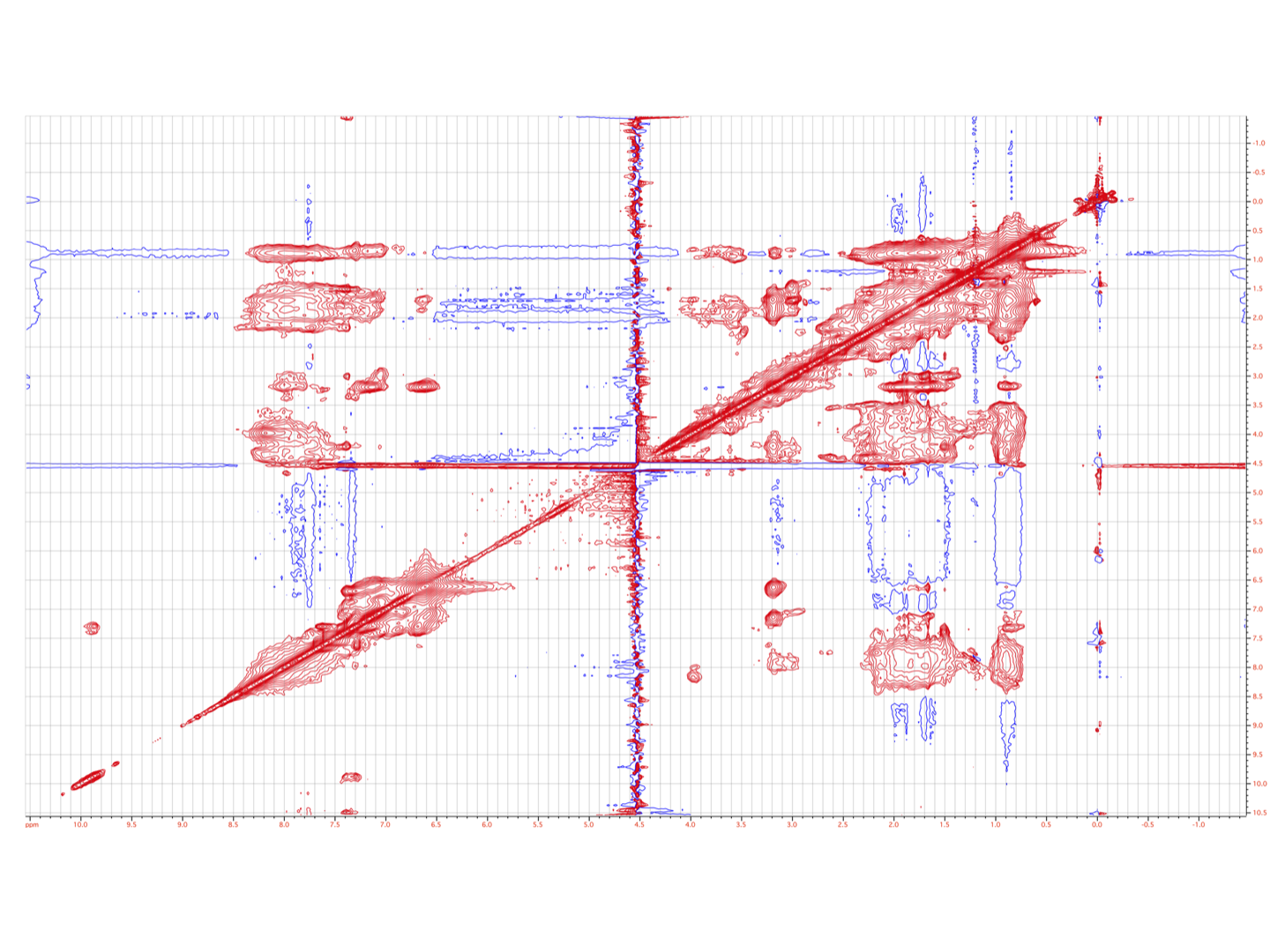

Supplement: Figure S3 — Full 2D NOESY NMR spectrum of 1 mM SP-B (1–25,63–78) in 150 mM SDS solution at pH 5 and 45°C. (TIFF) [file pone.0072821.s003.tiff]

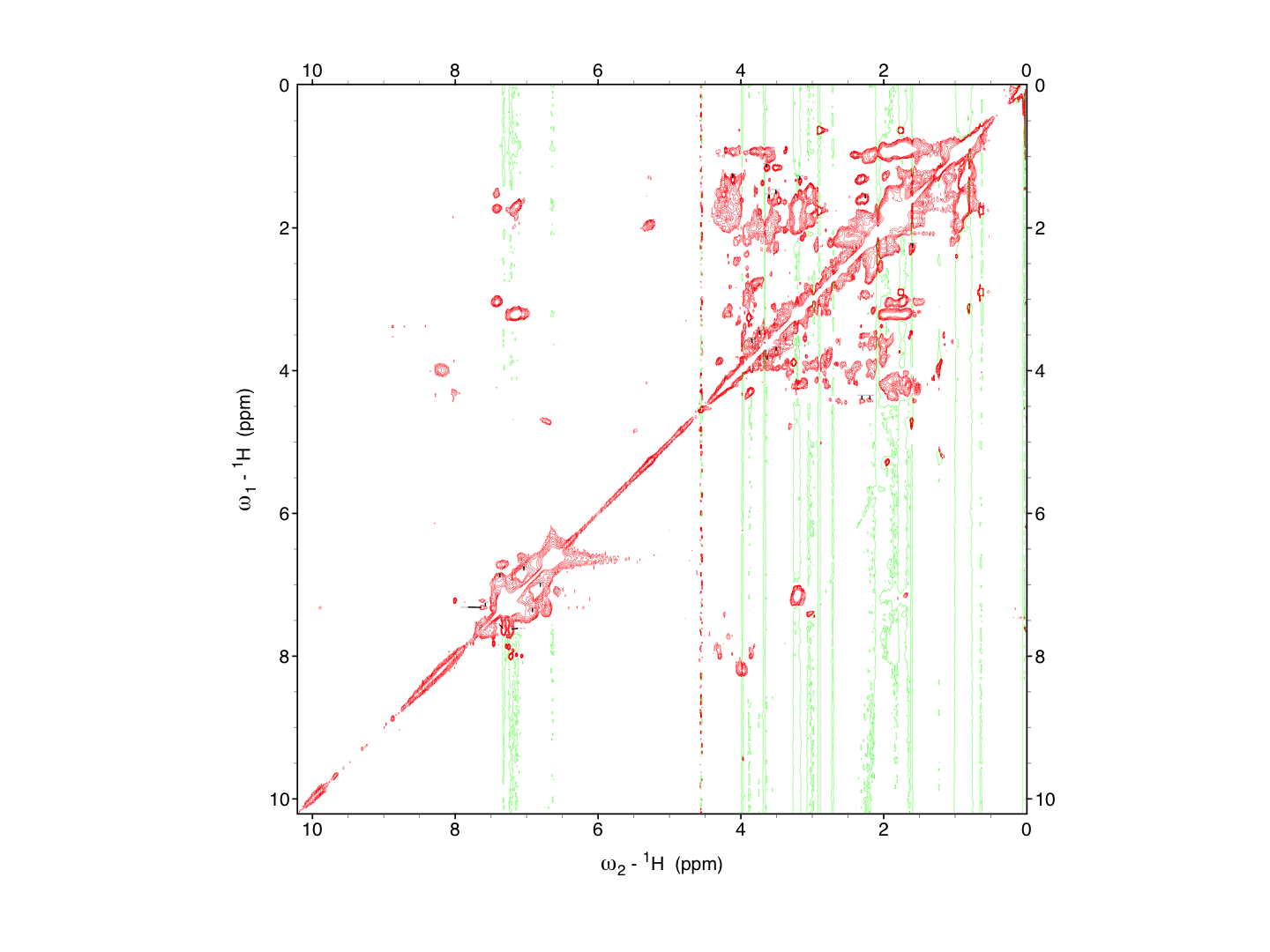

Supplement: Figure S4 — 2D NOESY NMR spectrum of SP-B (1–7) in 300 mM SDS solution at pH 5 and 45°C. (TIFF) [file pone.0072821.s004.tiff]

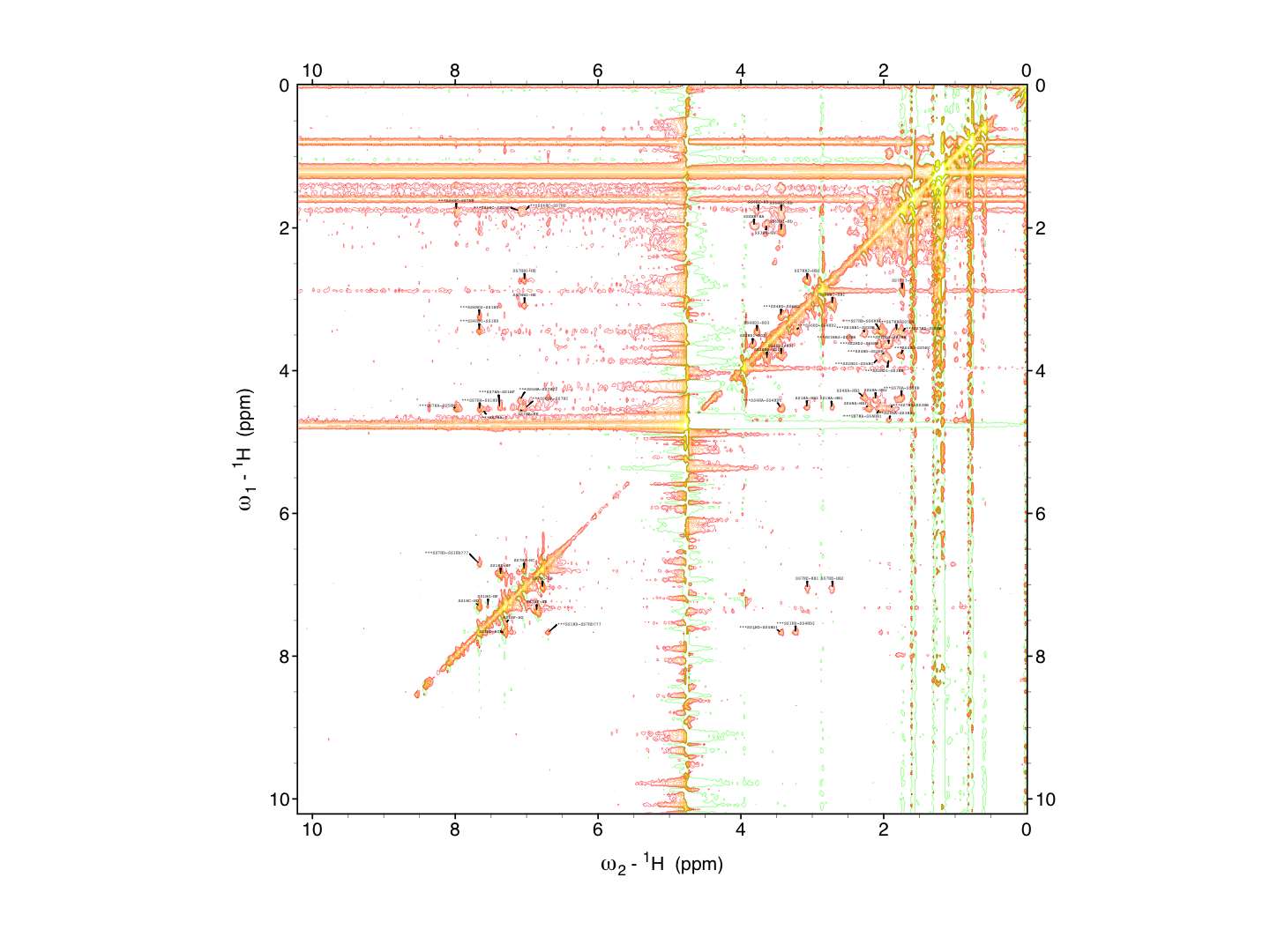

Supplement: Figure S5 — 2D TOCSY NMR spectrum of SP-B (1–7) in 300 mM SDS solution at pH 5 and 45°C. (TIFF) [file pone.0072821.s005.tiff]
